# Effect of an Internet-Based Pilates Telerehabilitation Intervention in People With Multiple Sclerosis: Protocol for a Randomized Controlled Trial

**DOI:** 10.2196/58026

**Published:** 2025-02-03

**Authors:** Andrea Tacchino, Michela Ponzio, Paolo Confalonieri, Letizia Leocani, Matilde Inglese, Diego Centonze, Eleonora Cocco, Paolo Gallo, Damiano Paolicelli, Marco Rovaris, Loredana Sabattini, Gioacchino Tedeschi, Luca Prosperini, Francesco Patti, Edoardo Sessa, Elisabetta Pedrazzoli, Mario Alberto Battaglia, Giampaolo Brichetto

**Affiliations:** 1 Scientific Research Area Italian Multiple Sclerosis Foundation Genoa Italy; 2 Multiple Sclerosis Center Fondazione Istituto Neurologico Carlo Besta Milan Italy; 3 Vita-Salute San Raffaele University Milan Italy; 4 Department of Neurorehabilitation Sciences Casa di Cura Igea Milan Italy; 5 Department of Neuroscience, Rehabilitation, Ophthalmology, Genetics, Maternal and Child Health University of Genoa Genoa Italy; 6 Istituto di Ricovero e Cura a Carattere Scientifico - Ospedale Policlinico San Martino Genoa Italy; 7 Neurology Unit Istituto di Ricovero e Cura a Carattere Scientifico Neuromed Pozzilli Italy; 8 Department of Systems Medicine Tor Vergata University Rome Italy; 9 Department of Medical Science and Public Health University of Cagliari Cagliari Italy; 10 Department of Neuroscience University of Padua Padua Italy; 11 Department of Traslational Biomedicine and Neurosciences University A Moro Bari Italy; 12 Don Carlo Gnocchi Foundation Milan Italy; 13 Unità Operativa Multiple Sclerosis Rehabilitation Istituto delle Scienze Neurologiche di Bologna Bologna Italy; 14 Department of Advanced Medical and Surgical Sciences University of Campania Luigi Vanvitelli Naples Italy; 15 Department of Neurosciences S Camillo-Forlanini Hospital Rome Italy; 16 Department of Medical and Surgical Sciences and Advanced Technologies University of Catania Catania Italy; 17 Unità Operativa Sclerosi Multipla Azienda Ospedaliero Universitaria Policlinico G Rodolico San Marco University of Catania Catania Italy; 18 Centro Neurolesi Bonino Pulejo Messina Italy; 19 Rehabilitation Service of Padua Italian Multiple Sclerosis Society Padua Italy; 20 Department of Life Science University of Siena Siena Italy; 21 Rehabilitation Service of Genoa Italian Multiple Sclerosis Society Genoa Italy

**Keywords:** exergame, MS-FIT, Pilates, Kinect, multiple sclerosis, exercise, physical activity, leisure time physical activity, Timed Up and Go

## Abstract

**Background:**

Physical activity (PA) has been recommended in multiple sclerosis (MS) to maintain good physical fitness and mental health, reduce the severity of symptoms and risk of relapse, and improve quality of life. Pilates has been suggested as an ideal PA to manage physical, cognitive, and psychological symptoms of MS and a useful method to maintain and improve balance and gait.

**Objective:**

This paper presents the protocol for a study that aims to evaluate the efficacy on the physical domain (specifically balance and gait) of a home-based, self-managed PA intervention delivered through the MS-FIT exergame (HELAGLOBE Società a responsabilità limitata). In addition, measures of cognitive performance, quality of life, and well-being will be considered.

**Methods:**

This is a 2-arm, multicenter, randomized controlled trial with 3 assessment points (baseline, 12 weeks postintervention, and 6 weeks follow-up). People with MS with mild disability, low risk of falling, preserved cognitive functions, and low anxiety and depression are potential eligible participants. The experimental group (MS-FIT) will self-administer the MS-FIT exergame at home in addition to their leisure-time physical activities. MS-FIT is an internet- and Pilates-based tool that uses the Microsoft Kinect Sensor V2. Participants in the control group will only have access to their leisure-time physical activities. Participants in the MS-FIT group will train at home with MS-FIT for 12 weeks and will be required to perform the exercises for a total of 30 minutes/day for at least 3 days/week. The primary outcome is the Timed Up and Go, a test designed to assess walking. We will also administer additional tests for motor function (visual analog scale 0-10, Timed 25-Foot Walk, Ambulation Index, 2-minute walk test, Twelve Item Multiple Sclerosis Walking Scale, Nine-Hole Peg Test), cognition (Brief International Cognitive Assessment for Multiple Sclerosis), fatigue (Modified Fatigue Impact Scale), quality of life (Multiple Sclerosis Quality of Life-54), well-being (Psychological Well-Being Scales), and PA (International Physical Activity Questionnaire and Minnesota Leisure Time Physical Activity Questionnaire). Acceptance and satisfaction with the intervention received (Client Satisfaction Questionnaire and an adapted version of the Tele-healthcare Satisfaction Questionnaire – Wearable Technology) and subjective impressions of changes in performance (Patients’ Global Impression of Change) will also be assessed.

**Results:**

Recruitment for the trial started on March 16, 2022, and the first participant was randomized the same day. Data analysis and results are expected to be published in 2025.

**Conclusions:**

Pilates has proven beneficial in several neurological diseases such as MS. With this study, we will provide evidence for the use in clinical practice of a digital tool for self-administered Pilates exercises at home as a complement to rehabilitation and for the continuity of care in MS.

**Trial Registration:**

ClinicalTrials.gov NCT04011579; https://tinyurl.com/2p9n4d2t

**International Registered Report Identifier (IRRID):**

DERR1-10.2196/58026

## Introduction

Multiple sclerosis (MS) is a chronic inflammatory neurodegenerative disease with autoimmune demyelinating lesions of the central nervous system and one of the most common causes of neurological disability in young adults. Clinically, MS progressively worsens over time, leading to cumulative physical disability, cognitive deficits, and neuropsychiatric and behavioral symptoms [1]. Physical impairments lead up to 85% of people with MS to complain of ambulation difficulties after typically 10-15 years of disease, and, after 20 years, over 66% of people with MS do not retain the ability to walk [1]. Several factors, such as weakness, spasticity, cerebellar ataxia, fatigue, and impaired attention, can cause imbalance and gait disorders [2,3]. They prevent people from performing daily activities properly and regularly, with negative effects on work status and social relationships. Rehabilitation is the main option to enhance recovery from disabling symptoms such as spasticity, ataxia, sensory loss, fatigue, pain, mood, and cognitive disorders [4]. More recently, people with MS have been recommended to engage in regular physical activity (PA).

PA, including leisure-time physical activity and exercise, comprises any bodily movement produced by skeletal muscles’ contraction that results in a substantial increase in energy expenditure over resting levels [5]. Increasing PA and reducing sedentary behavior are general recommendations to improve health outcomes for all people with chronic diseases [6]. For people with MS, PA maintains good physical fitness and mental health, prevents or reduces the severity of symptoms and the risk of relapses, and improves quality of life [7-9]. Based on current evidence and experts’ opinions, recent MS guidelines recommend “at least 150 minutes/week of exercise and/or 150 minutes/week of lifestyle PA” throughout the disease course [10]. However, Klaren et al [11] report that people with MS do not engage in sufficient PA amounts, with only approximately 20% of people with MS meeting recommended levels of moderate or vigorous daily activity compared with 40% of healthy participants. Although a recent survey study reported higher rates among people with MS, only 60% of the total sample met recommendations, with the lowest rate shown in the severely disabled group [12]. Furthermore, the benefits of PA may be limited if not incorporated into a structured, personalized, and supervised program.

Various mind-body fitness modalities, such as Pilates, yoga, Tai chi, and Qigong, are widely used by people who seek to achieve physical and mental health outcomes [13,14].

Pilates has been suggested as an ideal approach to managing physical, cognitive, and psychological symptoms of MS [15] and a popular alternative method for maintaining and improving balance and gait performance [16], as in other neurological conditions [17-19]. Indeed, it is a precise and controlled form of exercise that uses the body’s stabilizing muscles and is based on the principles of concentration, control, centering, flowing movement, precision, and breathing. If followed correctly, they can improve flexibility, strength, core stability, muscle control, breathing, and posture, and increase body awareness with less ground impact and joint stress [20,21]. The research findings support the therapeutic use of Pilates in the management of MS because it is a safe, active treatment method (few adverse effects) with high adherence (low dropout rate), and it can improve important meaningful functions (eg, balance, gait, physical-functional capacities, and cognition) [22].

A possible perspective for delivering Pilates interventions could benefit significantly from the proliferation of exergames and new technologies for telerehabilitation [23]. Playing exergames is a form of whole-body physical exercise [24] that requires users to complete assigned tasks aimed at improving physical fitness and promoting an active lifestyle [25]. Furthermore, exergames have been demonstrated to be comparable with traditional therapies, may be more enjoyable and acceptable, and may improve the engagement of people with MS in PA [26]. Exergame-based telerehabilitation solutions represent an efficient alternative method to overcome barriers that prevent people with MS from accessing regular long-term rehabilitation interventions (ie, transportation and working time) and to provide effective treatments in a setting, matching the patient’s circumstances (eg, at home), priorities (eg, during lunch break), and capabilities (eg, physical impairment) [27].

Recently, MS-FIT (HELAGLOBE Società a Relazioni Limitate), a Kinect-based playable exergame implementing Pilates exercises, has been developed by an Italian network of experts in the field of MS rehabilitation for future use in research and clinics [28]. It has been customized for people with MS, allows tailoring the intervention in order to potentiate the effects of an ongoing program, and has been conceived to be developed for asynchronous telerehabilitation purposes.

The aim of this study, promoted by the Italian Multiple Sclerosis Foundation, is to test in people with MS the efficacy on the mobility domain measured with the Timed Up and Go (TUG) test of a self-managed home intervention delivered through MS-FIT in addition to leisure-time physical activities, in comparison with leisure-time physical activities alone. Measures of balance, gait, upper limb functioning, PA, cognition, quality of life (QoL), well-being, acceptability, satisfaction with use, and adherence to the intervention will also be considered. In addition, a blood sample will be collected to investigate if genetic polymorphisms that are considered potential regulators of neural plasticity could influence the response to the proposed intervention [29].

Based on previous findings [30], we expect that the intervention, in addition to leisure-time physical activities, will be associated with a primary outcome of mobility (TUG) compared with leisure-time physical activities alone that are expected to worsen due to disease progression. Similar changes between groups are expected in secondary outcomes; furthermore, based on our previous findings [28], we expect high acceptability, satisfaction-to-use, and adherence to the MS-FIT intervention.

If results are positive, MS-FIT could be proposed in combination with rehabilitation to potentiate the effect of the rehabilitation intervention and ensure continuity of care for people with MS.

## Methods

### Study Design

We are conducting a multicenter 2-arm, superiority randomized controlled trial (RCT) with 3 assessment points (baseline, T0; postintervention at 12 weeks, T1; and follow-up at 6 weeks, T2). Participants will be allocated to the intervention (MS-FIT) or control group in a 1:1 ratio by simple randomization.

**Figure 1:**
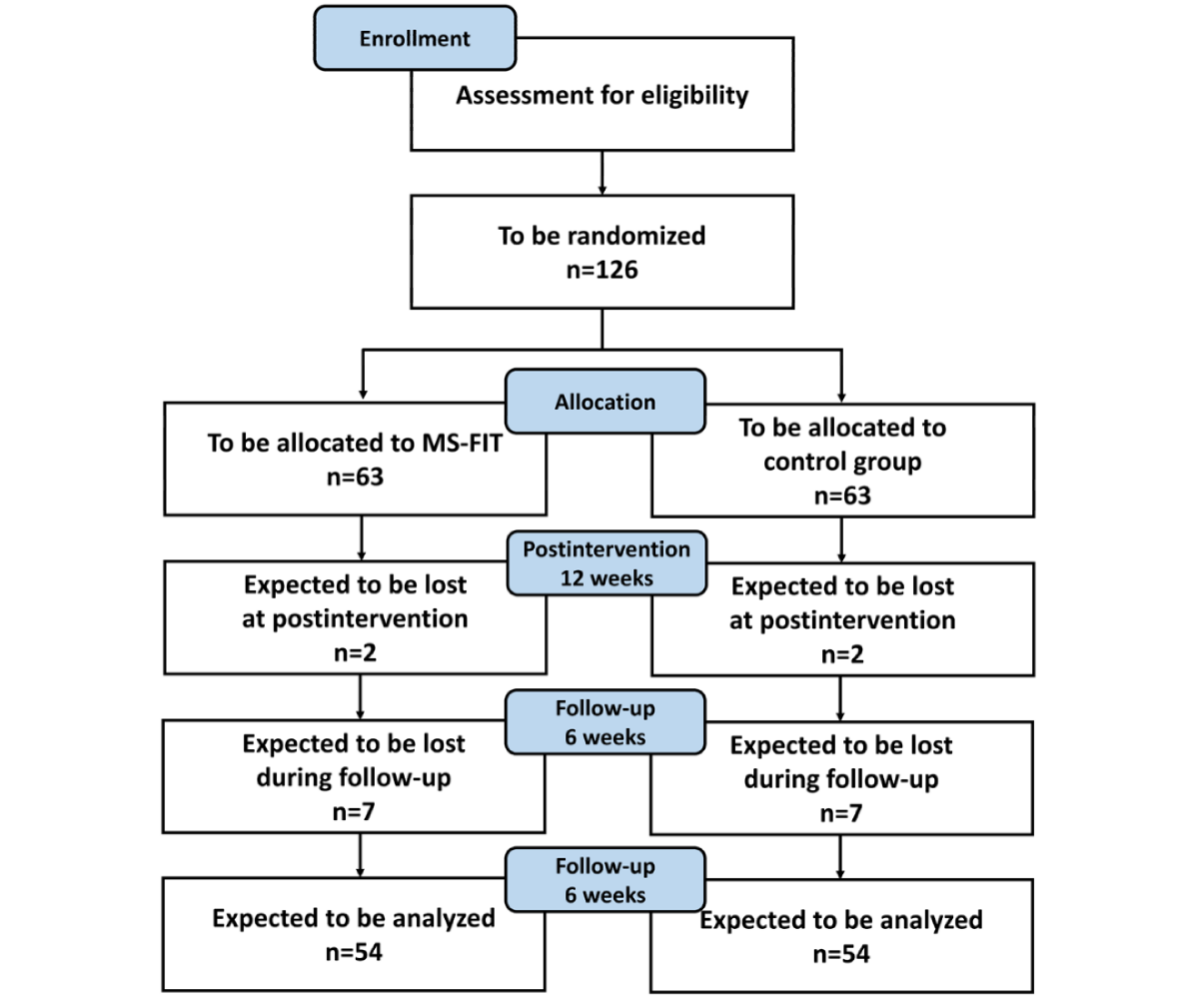
Study design.

Participants in the MS-FIT group will have access to the MS-FIT exergame for at-home self-administration [28] (Multimedia Appendix 1) in addition to their leisure-time physical activities. Participants in the control group will have the possibility to perform only their leisure-time physical activities. Exercise will not be allowed and rehabilitation treatments, except for speech therapy, sphincter rehabilitation, and psychological support, must be suspended. The study design is illustrated in Figure 1.

### Setting

Participants will be recruited among those followed by 14 Italian neurorehabilitation centers; specifically, at the Italian Multiple Sclerosis Society Rehabilitation Services of Genoa and Padua, the Multiple Sclerosis Center of IRCCS (Istituto di Ricovero e Cura a Carattere Scientifico) Foundation “Carlo Besta” Neurological Institute (Milan), the Vita-Salute San Raffaele University (Milan), the Department of Neuroscience, Rehabilitation, Ophthalmology, Genetics, Maternal and Child Health (DINOGMI) of the University of Genoa, the Neurology Unit of IRCCS Neuromed (Pozzilli), the Department of Medical Science and Public health of the University of Cagliari, Department of Translational Biomedicine and Neurosciences (DiBraiN) of the University A. Moro (Bari), the IRCCS Fondazione Don Carlo Gnocchi ONLUS (Milan), the Uosi Multiple Sclerosis Rehabilitation of the IRCCS Istituto delle Scienze Neurologiche of Bologna, Department of Advanced Medical and Surgical Sciences (DAMSS) of the University of Campania Luigi Vanvitelli (Naples), the Department of Neurosciences of the S. Camillo-Forlanini Hospital (Rome), the Department of Medical and Surgical Sciences and Advanced Technologies “G.F. Ingrassia” (DGFI) of the University of Catania, and the IRCCS Centro Neurolesi “Bonino-Pulejo” (Messina).

The assessments will be carried out in person at the several centers. The intervention with MS-FIT will be performed at home.

### Recruitment, Enrollment, and Randomization

People with MS along with mild disability, low risk of falling, preserved cognitive functions, and low anxiety and depression as reported in medical records are potential eligible participants. They will be contacted by telephone by the study manager of the related center (Multimedia Appendix 1), who will explain the study process in detail to ensure that patients understand the entire clinical trial; they will receive detailed information, including the purpose, procedures, follow-up content, data storage, benefits, and risks of the study, and will be given adequate time to consider participation. They will be informed that for the entire period of participation in the study, only leisure-time physical activities will be allowed, but not exercise and rehabilitation. They will also be informed verbally that their participation is voluntary and that they can withdraw their consent at any time without giving reasons and without depriving themselves of any treatment and care or other disadvantages.

Patients who agree to participate will be scheduled for a screening visit with a therapist (Multimedia Appendix 1). First, they will be asked to sign the written informed consent (Multimedia Appendix 2) after carefully reading and understanding the information about the study procedures and data security measures (including information on how to contact the study team in case of questions) provided to them. This approach is compliant with the General Data Protection Regulation and has been approved by the ethics committees.

After consent, recruited patients will proceed to the screening evaluation, where inclusion and exclusion criteria are assessed, and they will immediately receive feedback on whether or not they can participate in the study. Enrolled patients will be randomized by the study manager through the electronic case record form (eCRF) (Multimedia Appendix 1) into one of the 2 groups (ie, MS-FIT and control) and informed of the result of the assignment.

### Eligibility Criteria

Eligibility criteria were selected to ensure optimal fitness of the study sample with respect to the subsequent implementation of the MS-FIT intervention in the health care setting, to minimize the impact of confounding variables (eg, rehabilitation outcomes), and to ensure comparability with other studies on similar interventions.

#### Inclusion Criteria

The study includes people with a diagnosis of MS according to McDonald’s criteria [31] and aged ≥18 years; all disease courses are eligible (relapsing-remitting, primary progressive, and secondary progressive) [32]. Patients will be included if their disability level based on the Expanded Disability Status Scale (EDSS) [33] is between 2 and 4. Indeed, we expect that in people with MS with a lower EDSS, the proposed intervention might not be effective and other forms of PA based mainly on exercise should be considered. For people with MS with a higher EDSS, Pilates could be recommended as a complement but not as a replacement for rehabilitation; also, for this reason, we considered it unethical to include patients who should suspend their treatments for the aims of the study. Furthermore, asynchronous telerehabilitation tools for self-administered balance training should be proposed when the patient’s ability to maintain balance safely is preserved (eg, pregnant women will be excluded until after giving birth). In any case, we included patients with preserved balance evaluated with the Berg Balance Scale score >46 [34].

To ensure the ability to use the MS-FIT exergame, normal cognitive functioning determined by a Mini-Mental Status Examination score >24 [35] is required; a Hospital Anxiety and Depression Scale score <10 in the 2 subsets of anxiety and depression [36] could prevent a loss of adherence due to mood disorders.

#### Exclusion Criteria

The study excludes individuals who have received at least 1 rehabilitation treatment (except speech therapy, sphincter rehabilitation, and psychological support) in the last month before being contacted. Other exclusion criteria are visual deficits that could compromise the use of the MS-FIT exergame and relapses in the last 3 months. Participants with relapses during the period of involvement in the study will be considered dropouts.

All hardware and software components will be provided to the participants in the MS-FIT group; training provided by the study manager will ensure ease of use. For this reason, technology will not be an exclusion criterion.

### Intervention

All participants will be allowed to continue their leisure-time physical activities.

Participants in the MS-FIT group will receive the MS-FIT exergame [28]. They will be required to train at home with MS-FIT for 12 weeks by performing the exercises for a total of 30 minutes/day for at least 3 days/week.

While gaming, participants will choose their favorite and most useful exercises; the difficulty of the exercise will increase based on the user’s performance. In this way, the exercise selection and the reward structure will ensure the personalization of the intervention.

Before starting the intervention, each participant will be trained in the use of the tool by the study manager.

Participants in the control group will not receive MS-FIT; however, as a reward for their participation, we will offer them the MS-FIT intervention at the end of their study involvement.

All participants will be assessed at T0, T1, and T2.

### Assessment

The assessment will consist of clinical tests measuring the domains most relevant to MS. The primary outcome will always be administered first; the order of the secondary outcomes will be randomized across participants; however, the same order will be maintained for the individual participant. We assume that the administration of all tests will take, on average, about 60 minutes.

Multimedia Appendix 3 summarizes when the different outcome measures should be collected.

#### Primary Outcome

The primary outcome of the study is TUG [37], commonly used to measure dynamic balance and to assess activity limitations by examining the patient’s ability to ambulate and perform transfers. The TUG was originally created to predict the risk of falling in geriatric patients.

At the “go” signal, the participant must rise from a chair, walk 3 m, turn around, return to the chair, and sit down as quickly but safely as possible. The participant starts with the back against the chair and the arms resting on the armrests and is timed from the moment he or she lifts the pelvis from the chair until he returns with the pelvis on the chair [34]. The participant wears his or her regular footwear and uses his or her customary walking aid (none, cane, or walker); however, due to the eligibility criteria adopted in this study, participants will not use assistive devices for walking. No physical assistance is given.

#### Secondary Outcomes

Secondary outcomes will evaluate the effects of the intervention on the following:

1. Motor function: visual analog scale (VAS; 0-10) for balance subjective disability improvement [38], Timed 25-Foot Walk (T25FW) [39], Ambulation Index [40], 2-minute walk test (2MWT) [41], Twelve Item Multiple Sclerosis Walking Scale (MSWS-12) [42,43], and Nine-Hole Peg Test (9HPT) [44].
2. Cognition: Brief International Cognitive Assessment for Brief International Cognitive Assessment for Multiple Sclerosis (BICAMS), which consists of the Symbol Digit Modalities Test (SDMT), the California Verbal Learning Test-II (CVLT-II), and the Brief Visuospatial Memory Test-Revised (BVMT-R) [45].
3. Fatigue: Modified Fatigue Impact Scale (MFIS) [46,47].
4. QoL: Multiple Sclerosis Quality of Life-54 (MSQoL-54) [48,49].
5. Well-being: Psychological Well-Being Scales (PWB) [50,51].
6. PA: International Physical Activity Questionnaire (IPAQ) [52,53], the Minnesota Leisure-Time Physical Activity Questionnaire [54] which provides a general evaluation of the total energy expenditure in leisure time physical activity.
7. Perceived intervention effect: 7-point Patients’ Global Impression of Change (PGIC; from 1=“no change or worse” to 7=“a great deal better”) scale.

#### Technology

Furthermore, acceptance of the used technology will be evaluated with an ad-hoc questionnaire on patients’ expectations; satisfaction with the intervention will be evaluated with the Client Satisfaction Questionnaire (CSQ-8) [55,56] and the Tele-healthcare Satisfaction Questionnaire (TSQ-WT) [57].

#### Blood Sample

If the participant consents, a peripheral blood sample will be collected at T0 in vacutainer tubes containing ethylenediaminetetraacetic acid. According to previous studies [29], participants will be divided into subgroups with respect to polymorphism. All blood samples will be genotyped for a total of 55 genetic polymorphisms of 23 potential regulators, such as Homer1, AKT1, RAPTOR, D2R, GAPD, CHAT, p53, BRCA2, LIG4, XRCC5, CYP3A4, NBS1, MDM2, CNR1, ATTn, CNR2, GRIN1, GRIN2B, TRPV1, FAAH, COMPT, and BDNF.

For example, CNR1 region containing AAT repetitions will be amplified by polymerase chain reaction from genomic DNA; sequencing products will be purified using a rapid, high-performance dye-terminator removal kit and will be subjected to electrophoresis; AAT repetitions will be counted on the resulting electropherograms (short AAT: homozygous or heterozygous for an allele with ≤11 repeats of AAT triplets; long AAT: homozygous for an allele with ≥12 repeats of AAT triplets). For the TRPV1 and FAAH, the iPLEX Gold technology and MassARRAY high-throughput DNA analysis will be performed.

The blood sample will be collected early in the morning after awakening (8:00 AM). To synchronize the sample with lifestyle variables, participants were requested to avoid excessive physical activity in the last 3 days before blood sampling, to sleep for 7-8 hours the night before the study, to avoid starvation, and to have a normal breakfast in the morning [29]. The samples will be analyzed in the center of DC.

#### Other Measures

Adherence to the intervention will be provided by the number of sessions actually performed as automatically recorded by the MS-FIT tool and the number of dropouts.

Safety will be assessed by the number of potential issues (ie, number of falls). The severity of adverse events will be graded according to the Common Terminology Criteria for Adverse Events v 4.0 on a 5-point scale (grade 1, mild discomfort; grade 2, moderate discomfort; grade 3, severe inability; grade 4, life-threatening or disabling; grade 5, death) and reported in detail on the eCRF. A grade higher than grade 2 will be considered as a condition for dropout.

We will also collect information on the pharmacological treatments (specific and nonspecific for MS) followed by participants, comorbidities, and the type of PA practiced during participation in the study.

### Criteria for Premature Withdrawal

In addition to withdrawal of consent at any time, the study manager at each site may also consider as criteria for premature withdrawal any medical conditions that could jeopardize the safety of the patient if she or he continues the study, changes in the disease treatment during the study, and noncompliance of the participants to the study procedures.

### Sample Size

The sample size was calculated by referring to the TUG change after a Pilates intervention found by Kalron et al [30] in a group of people with MS. The authors found no difference between the group receiving the Pilates intervention and the control group receiving an intervention of physical therapy. The Pilates group improved the TUG performance by about 1.8 seconds, which is clinically relevant for people with MS. Considering a variability of about 3.4 seconds, a power of 80%, a significance level (2-sided) of 5%, and a potential loss of 15% of patients at follow-up, the estimation of the necessary sample size consists of approximately 63 participants for the experimental group (a total of 126).

### Data Collection and Management

Data entry, including quality checks and double entry validation, will be performed through eCRF (Multimedia Appendix 1). Missing data from the unit record will be compared with the corresponding handwriting case record form and corrected accordingly.

### Statistical Analysis

Descriptive statistics will be used to evaluate differences between the 2 groups of participants. The study hypotheses will be tested using an intention-to-treat analysis, where all consenting patients who were randomized during the accrual period will be included in the analysis.

Continuous data will be summarized using count, mean, median, SD, and IQRs, whereas categorical measures will be described using frequencies. The assumption of normality will be tested with the Shapiro-Wilk test. Pre-post effects within groups will be investigated using the paired *t* test or the Wilcoxon matched-pairs signed-rank test. Between-group proportions will be compared by chi-square or Fisher exact test, while between-group comparisons for continuous variables will be done using either the unpaired 2-sided *t* test or the 2-sided Wilcoxon 2-sample test for nonnormal data. Correlations will be computed using Pearson or Spearman coefficients.

The primary analysis will be performed by comparing mean changes from T0 to T1 in the primary and secondary study outcomes. In particular, the MS-FIT group will be compared with the control group with analyses of covariance. The model includes change as the dependent variable, with the intervention group as the main effect and baseline and number of sessions as additional covariates. Finally, generalized estimating equation models will be performed to assess the persistence of the intervention effect; here the mean effect of the intervention at the time points T0, T1, and T2 on all outcomes, adjusted for the baseline value, will be evaluated between the 2 groups.

Differences in the outcome measures between subgroups defined by different polymorphisms will be analyzed using the analysis of variance or the Kruskal-Wallis test, as appropriate.

Statistical analysis will be performed using STATISTICA 7.1 software (StatSoft GmbH). Significance will be recognized for *P*<.05.

### Ethical Considerations

This trial is conducted in accordance with the protocol (version 1, July 27, 2018), the Declaration of Helsinki, and good clinical practice. It has been approved by the Ethics Committee of San Martino Hospital (134/2018) and registered before patient enrollment at ClinicalTrials.gov (NCT04011579).

Any change to this protocol must be agreed upon by all the study investigators. Each amendment must be signed by the principal investigator at each site, and amendment forms will be submitted to the local Ethics Committee for approval. The amendment will be updated in the clinical trial registry.

Participants will be asked to sign the written informed consent, and their data will be deidentified. Participants will not receive any compensation for participating in the study.

A formal audit process at the end of the study is proposed for this trial.

### Patient and Public Involvement

Patients and the public were not involved in the design, conduct, reporting, or dissemination plans of our research, although patient feedback was an important source for the development and improvement of the MS-FIT tool investigated in this trial [28]. Published results will be disseminated to study participants upon request.

### Dissemination

The results of this RCT study will be disseminated. Dissemination includes communication and promotion of the project activities and results to participants, people with MS, and their stakeholders (eg, caregivers, health care professionals, and the scientific community). Dissemination activities will begin at launch and will continue throughout the duration of the study. Specific activities include dissemination through materials such as flyers and videos to be distributed through the internet, social networks, forums, or at local, national, and international events, and specific meetings with end users; dissemination through presentation of results in national and international peer-reviewed scientific journals; dissemination through presentation of research results at major national and international health care conferences and annual meetings; and dissemination through the ClinicalTrials website registry.

## Results

Recruitment for the trial started on March 16, 2022, and the first participant was randomized on the same day. As of March 2022, we enrolled 126 participants who received a T0 visit; up to now, 108 received a T1 visit and 103 received a T2 visit. Data analysis and results will be published in early 2025.

## Discussion

### Summary

Cumulative data suggest that the Pilates method has proven beneficial in several neurological diseases and may be a PA tool for people with MS. A recent review [22] in MS showed that Pilates improves balance, gait, physical-functional conditions (muscle strength, core stability, aerobic capacity, and body composition), and cognitive functions; in contrast, fatigue, QoL, and psychological function did not show a clear improvement. Furthermore, high adherence (average adherence ≥80%) and few adverse effects were reported. For these reasons, future research is needed to develop clinical protocols that can maximize the therapeutic effects of Pilates for people with MS. In this context, novel devices and technologies for home-based interventions could provide solutions to overcome limitations due to urban barriers (eg, transportation), daily activities (eg, working time), and clinical conditions (eg, level of disability) and to ensure continuity of care. Therefore, practicing Pilates at home through eHealth tools could help people with MS to regularly engage in PA and to successfully maintain their physical, cognitive, and emotional status.

Here, we describe the RCT protocol designed to test the efficacy of a home-based PA intervention delivered through the MS-FIT exergame on balance and gait. In addition, we will also assess the effects on cognition, QoL, and well-being.

The present RCT protocol was designed taking into account previous studies in the MS scientific literature in terms of the number of weeks of intervention, weekly frequency, session duration, outcomes, setting, and control group [22]. Because only one study evaluated long-term monitoring after the intervention [58] and it is unclear whether the obtained results are maintained, as an additional element of novelty, we propose to schedule a follow-up assessment in order to define whether the effects of Pilates persist.

### Limitations

The first limitation of this study is that we will only have evidence of the effectiveness of a Pilates exergame for people with MS with mild disability. However, even people with MS at more advanced disease stages benefit from regular PA to maintain fitness, prevent pain and secondary complications of inactivity, and treat or reduce symptoms. Thus, as specific prescriptions should be tailored to clinical conditions, a dedicated trial for patients with a higher level of disability should be considered. Second, due to the discontinuation of the Kinect Sensor V2 adopted in MS-FIT, for the successful translation of the exergame into clinical practice, a new compatible commercial sensor will be identified, and the app will be adapted; Microsoft Azure Kinect and Mentor Age (Wita Società a responabilità limitata) are valid and comparable systems already taken into account by other researchers [28,59,60]. Third, although the MS-FIT tool was not developed to pursue rehabilitation purposes, it would be helpful to analyze the MS-FIT intervention as a complement to the work of health care professionals and evaluate if it is able to potentiate the effect of a rehabilitation intervention, possibly also in people with MS with a higher level of disability. Finally, direct evidence, such as MRI acquisition, will not be considered to reveal which microstructural changes are due to the MS-FIT intervention.

### Future Directions and Conclusions

MS-FIT is a usable and accepted digital health tool for telerehabilitation implementing Pilates exercises and aims at supporting the self-administration of Pilates training at home. This study will provide evidence for the use of the MS-FIT tool in clinical practice as a complement to rehabilitation and for the continuity of care of people with MS. However, a feasibility protocol involving patients with moderate disability (EDSS between 4 and 6) should be designed and tested, followed by a multicenter trial that assesses the effectiveness of the tool and paves the way for more extensive use in MS.

## Appendix

Multimedia Appendix 1 Description of the digital tools used in the study and of the personnel involved in the study activities.

Multimedia Appendix 2 Original Italian version of the information sheet and its English translation.

Multimedia Appendix 3 The collection of the outcome measures occurs at different time points depending on the considered measure.

## Abbreviations

2MWT: 2-minute walk test
9HPT: 9-Hole Peg Test
BICAMS: Brief International Cognitive Assessment for Multiple Sclerosis
CSQ-8: Client Satisfaction Questionnaire
eCRF: electronic case record form
EDSS: Expanded Disability Status Scale
IPAQ: International Physical Activity Questionnaire
IRCCS: Istituto di Ricovero e Cura a Carattere Scientifico
LTPA-Q: Leisure Time Physical Activity Questionnaire
MFIS: Modified Fatigue Impact Scale
MS: multiple sclerosis
MSQoL-54: Multiple Sclerosis Quality of Life-54
MSWS-12: 12-Item Multiple Sclerosis Walking Scale
PA: physical activity
PGIC: Patients’ Global Impression of Change
PWB: Psychological Well-Being Scales
QoL: quality of life
RCT: randomized controlled trial
T25FW: Timed 25-Foot Walk
TSQ-WT: Tele-healthcare Satisfaction Questionnaire – Wearable Technology
TUG: Timed Up and Go
VAS: visual analog scale

## Data Availability

The data sets generated and analyzed during this study will be available from the corresponding author on reasonable request.
