# Supplementary material for: Effect of an Internet-Based Pilates Telerehabilitation Intervention in People With Multiple Sclerosis: Protocol for a Randomized Controlled Trial
Source: JMIR Res Protoc. 2025 Feb 3;14:e58026. doi: 10.2196/58026 (PMC11833266; doi:10.2196/58026)
Supplement: Multimedia Appendix 1 [file resprot_v14i1e58026_app1.docx]

**MS-FIT Tool**

The MS-FIT exergame is a game-based individual interactive training tool developed with the Unity 3D game engine software (Unity Technologies). The hardware configuration consists of 4 devices: a minicomputer that implements the exergame (e.g, Intel Next Unit Computer), a monitor with an HDMI connector, a power lead controller to access the exergame and navigate the menus, and a Microsoft Kinect Sensor version 2. The exergame can be played with or without an internet connection.

MS-FIT implements Pilates exercises appropriately adapted to the requirements of MS. The usability of the tool has been successfully tested in a previous work. The Microsoft Kinect Sensor V2 allows the user to interact via gestures and voice commands that ensure better access, participation, and benefit for people with disabilities such as PwMS.

MS-FIT implements Pilates exercises identified for safe execution via a digital tool; the exercises aim to train breathing, posture, and balance. A teacher-avatar verbally explains and visually demonstrate the exercise that the user will have to perform. The Kinect will record the user’s movements and, consequently, will display the user’s avatar on the screen. The level of accuracy in the execution movements will determine the rewards for the user and, ultimately, access to new levels of difficulty.

MS-FIT is thought to be used without the synchronous supervision of the therapist.

**Electronic case record form (eCRF)**

For the aims of the study, an electronic case record form (eCRF) was designed and developed. The eCRF is a web-based, password-protected data management system (provided by Nubilaria srl, Italy) developed for the data entry of the papery data collected by authorized and trained personnel. The eCRF implements the randomization sequence previously generated using the routines of the website <http://www.random.org> and will be blind to the personnel involved in the study, except for one FISM researcher who prepared the sequence and will not be involved in the subsequent research.

**Personnel**

For each center, two healthcare professionals with a specific background in MS, namely the study manager (the principal investigator of the center who will be a physiatrist or neurologist) and a therapist, will be involved in the study activities. They do not necessarily have to be certified in Pilates; however, during a dedicated training, they will be introduced to the principles of Pilates.

The 14 study managers will perform recruitment, enrolment, randomization, digital data entry, and delivery of the MS-FIT tool to participants. To this end, they will receive dedicated online training with a specific focus on the use of the eCRF and the MS-FIT exergame. Through the eCRF, they will randomize participants, edit the study data and audit for data quality (database validation checks on missing data, out-of-range values, illogical entries and invalid responses).

Moreover, they will deliver the MS-FIT tool to participants randomized into the MS-FIT group and train them with a full description of its concept and rules, provided that they are autonomous in assembling, calibrating and using it.

The 14 therapists will perform the assessment at the 3 time points (i.e. T0, T1, and T2) and, for this purpose, will be trained in the administration of the tests in order to ensure uniformity of the methods implementation between the different centers; a dedicated training will be held in Milan before the start of the study. These assessors will be blinded to the group assignment.

At the end of the study, an external monitor will be involved to monitor and review the data entered by each site in the eCRF; data queries will be raised if necessary by the monitor and fixed by the study managers before starting the data analysis.
