# Supplementary material for: Effect of an Internet-Based Pilates Telerehabilitation Intervention in People With Multiple Sclerosis: Protocol for a Randomized Controlled Trial
Source: JMIR Res Protoc. 2025 Feb 3;14:e58026. doi: 10.2196/58026 (PMC11833266; doi:10.2196/58026)
Supplement: Multimedia Appendix 2 [file resprot_v14i1e58026_app2.doc]

**FOGLIO INFORMATIVO**

***“Multiple Sclerosis Fitness Intervention Training with Pilates exercises (MS-FIT)”***

Gentile Signore/a,

Le è stato proposto di prendere parte ad uno studio che verrà realizzato presso il Servizio di Riabilitazione AISM Liguria dall’area ricerca scientifica della Fondazione Italiana Sclerosi Multipla. Lo scopo di questo studio multicentrico è valutare l’effetto di un intervento di attività fisica con esercizi di Pilates in persone con SM (PcSM) autosomministrato a domicilio tramite il sistema MS-FIT basato su Kinect.

Recentemente un metodo alternativo molto popolare per il mantenimento e miglioramento di equilibrio e cammino è il Pilates, una tecnica di esercizio basata sulla stabilizzazione dei muscoli del corpo e su un approccio olistico che tramite la corretta esecuzione di sei principi fondamentali (concentrazione, controllo, centramento, fluidità, precisione, respirazione) aumenta la consapevolezza corporea diminuendo l’impatto al suolo e lo stress articolare. Gli esercizi di Pilates possono essere eseguiti a vari livelli di intensità a seconda delle capacità della persona. Nonostante la mancanza di evidenza scientifica a supporto dell’efficacia del Pilates, molti professionisti stanno promuovendone l’uso come strategia di trattamento nel caso di ictus o negli anziani.

Recentemente è cresciuto anche l’interesse per l’utilizzo del Pilates nella SM. Tuttavia, anche in questo caso, si hanno poche evidenze scientifiche nonostante l’interesse sia forte e vengano riportati miglioramenti in equilibrio e deambulazione da parte delle PcSM che lo praticano costantemente.

Per questa ragione attraverso il progetto MS-FIT si vuole testare se gli esercizi di Pilates, rilasciati tramite un sistema basato su Microsoft Kinect Motion Controller Xbox, può essere un buon metodo per promuovere l’attività fisica, l’integrazione sensorimotoria e la stimolazione cognitiva. In tal modo, questo pseudo-Pilates potrebbe rappresentare un’opzione di trattamento per migliorare la fatica, l’equilibrio e la capacità di camminare delle PcSM; il pseudo-Pilates potrebbe essere così suggerito dal clinico come un’attività fisica da essere integrata nella vita quotidiana.

**Obiettivi**

Gli obiettivi specifici di questo studio sono:

1. Uno studio di fattibilità per rifinire aspetti tecnici del sistema MS-FIT basato su Kinect, valutare l’accettabilità e la soddisfazione all’uso da parte di PcSM, per valutare il processo di reclutamento, l’aderenza all’intervento, il numero di drop-out e identificare potenziali problematiche, per valutare le necessità in termini di risorse umane per il successivo studio randomizzato e controllato, per stimare l’effetto dell’intervento e la sua varianza necessari per calcolare l’appropriata grandezza del campione per l’RCT.
2. Uno studio randomizzato e controllato per valutare l’effetto di un intervento di attività fisica con esercizi di Pilates in PcSM autosomministrato a domicilio tramite il sistema MS-FIT.

**Soggetti**

Un campione di 56 PcSM per lo studio di fattibilità e di 126 PcSM per lo studio randomizzato e controllato saranno reclutati dalle unità cliniche partecipanti. Le PcSM reclutate per lo studio di fattibilità non parteciperanno allo studio randomizzato e controllato.

Saranno inclusi PcSM di ambo i sessi con età maggiore di 18 anni, con diagnosi di Sclerosi Multipla, livello di disabilità misurato con la scala Expanded Disability Status Scale (valori da 2 a 4), livello di ansia e depressione misurato con la Hospital Anxiety and Depression Scale con valore < 10 nei due sottoset di ansia e depressione, l’equilibrio misurato con la Berg Balance Scale (valore > 46), stato cognitivo con il Mini-Mental State Examination (valore > 24), almeno un mese senza essere stati trattati con riabilitazione. Tutti i soggetti devono aver firmato il consenso informato.

Saranno esclusi tutti i soggetti che presentano ricadute negli ultimi tre mesi e che presentino deficit visivi che potrebbero compromettere l’uso di MS-FIT.

**Valutazioni e Trattamenti previsti**

Per lo studio di fattibilità le valutazioni verranno effettuate prima dell’inizio (PRE) e alla fine delle sei settimane di trattamento (POST). Per lo studio randomizzato e controllato le valutazioni verranno effettuate prima dell’inizio (PRE) e al termine delle dodici settimane di trattamento (POST) e dopo sei settimane dalla valutazione POST (follow-up, FU).

Sia lo studio di fattibilità che lo studio randomizzato e controllato prevedono due gruppi: il gruppo di controllo a cui è richiesto di continuare ad eseguire le normali attività fisiche che eventualmente già eseguono per tutta la durata delle 6 (trattamento; studio di fattibilità) o 12 + 6 (trattamento + follow-up; studio randomizzato e controllato) settimane; al gruppo sperimentale oltre a quanto richiesto al gruppo di controllo si richiede di eseguire gli esercizi di MS-FIT per almeno 3 volte alla settimana per un totale di 30 minuti a sessione (anche distribuiti durante il giorno con uno slot temporale minimo di 10 minuti) per le 6 (studio di fattibilità) o 12 (studio randomizzato e controlalto) settimane.

La somministrazione di MS-FIT verrà autogestita a domicilio dal partecipante a cui verrà preventivamente rilasciata la piattaforma Xbox One and Microsoft Kinect 2.0. La spiegazione delle parti del sistema e il training al suo utilizzo verranno effettuate prima del rilascio.

Per entrambi i gruppi non sarà possibile eseguire trattamenti riabilitativi eccetto la riabilitazione sfinterica e foniatrica e il supporto psicologico.

L’assegnazione al gruppo sperimentale o di controllo avverrà secondo procedure computerizzate random.

Ai partecipanti allo studio randomizzato e controllato verrà eseguito un prelievo di sangue in occasione della valutazione PRE.

**Raccolta informazioni**

Verrà realizzata una CRF elettronica per la raccolta di: dati clinici e demografici relativi ai soggetti reclutati; risultati dei test/questionari somministrati di cui sopra nel dettaglio.

I dati raccolti saranno resi anonimi mediante un codice e potranno essere accessibili solo ai responsabili dei gruppi di ricerca di ciascun partner partecipante al progetto.

**Copertura assicurativa**

La copertura assicurativa per il progetto è stata stipulata con la compagnia QBE, con numero di polizza 063 0000483, con valenza dalle ore 24.00 del 19/11/2022 alle ore 24.00 del 31/07/2023.

Il Massimale per soggetto è di 1000000 di euro mentre Massimale Aggregato per l'intera durata della Sperimentazione Clinica è di 7500000 di euro.

Il Termine per la manifestazione dei danni cagionati ai soggetti della sperimentazione è 24 mesi dal 31/07/2023 mentre il Termine per la presentazione delle richieste di risarcimento è 36 mesi dal 31/07/2023.

#### Centri partecipanti allo studio.

- Fondazione Italiana Sclerosi Multipla (FISM), Genova
- IRCCS Istituto Neurologico C. Besta, Milano
- Istituto di Neurofisiologia Sperimentale, Ospedale San Raffaele, Milano
- Università di Genova, Genova
- IRCCS Neuromed, Pozzilli, Isernia
- Università di Cagliari, Cagliari
- Università di Padova, Padova
- Università di Bari, Bari
- IRCCS Fondazione Don C. Gnocchi, Milano
- Università di Bologna, Bologna
- Università di Napoli, Napoli
- UOC Neurologia, Osp. S. Camillo-Forlanini, Roma
- Università of Catania, Catania
- IRCCS Centro Neurolesi “Bonino Pulejo”, Messina

**CONSENSO INFORMATO**

Io sottoscritto/a

COGNOME ____________________________________________ NOME__________________________________________

ACCONSENTO DI PARTECIPARE ALLO STUDIO

Firma del paziente _______________________________________________________ Data ______________________

Firma del medico__ _______________________________________________________ Data ______________________

**INFORMATION SHEET**

***“Multiple Sclerosis Fitness Intervention Training with Pilates exercises (MS-FIT)”***

Dear Sir/Madam,

You have been asked to take part in a study that will be carried out at the AISM Rehabilitation Service of Liguria by the scientific research area of ​​the Italian Multiple Sclerosis Foundation. The aim of this multicenter study is to evaluate the effect of a physical activity intervention with Pilates exercises in people with MS (PwMS) self-administered at home via the Kinect-based MS-FIT system.

Recently, a very popular alternative method for maintaining and improving balance and walking is Pilates, an exercise technique based on the stabilization of the body's muscles and on a holistic approach that, through the correct execution of six fundamental principles (concentration, control, centering, fluidity, precision, breathing), increases body awareness by decreasing the impact on the ground and joint stress. Pilates exercises can be performed at different levels of intensity depending on the individual’s abilities. Despite the lack of scientific evidence supporting the effectiveness of Pilates, many professionals are promoting its use as a treatment strategy for the stroke survivors or the elderly.

Recently, interest in the use of Pilates in MS has also grown. However, even in this case, there is little scientific evidence despite the strong interest and improvements in balance and walking reported by PwMS who practice it regularly.

For this reason, through the MS-FIT project we want to test whether Pilates exercises, released through a system based on Microsoft Kinect Motion Controller Xbox, can be a good method to promote physical activity, sensorimotor integration and cognitive stimulation. In this way, it could represent a treatment option to improve fatigue, balance and walking ability of PwMS; it could thus be suggested by the clinician as a physical activity to be integrated into daily life.

**Objectives**

The study aims are:

1. A feasibility study to refine technical aspects of the Kinect-based MS-FIT tool, to evaluate its acceptability and satisfaction by PwSM, to evaluate the recruitment process, adherence to the intervention, the number of drop-outs and identify potential issues, to assess the human resource needs for the subsequent randomized controlled trial (RCT), to estimate the effect of the intervention and its variance necessary to calculate the appropriate sample size for the RCT.
2. A RCT to evaluate the effect of a physical activity intervention with Pilates exercises in PwSM self-administered at home via the MS-FIT system.

**Subjects**

A sample of 56 PwSM for the feasibility study and 126 PwSM for the RCT will be recruited from the participating clinical units. PwSM recruited for the feasibility study will not participate in the RCT.

Adult PwMS of both sexes with a diagnosis of MS, level of disability measured with an Expanded Disability Status Scale score ​​in the range 2-4, level of anxiety and depression measured with the Hospital Anxiety and Depression Scale with a score < 10 in the two subsets of anxiety and depression, balance measured with a Berg Balance Scale score > 46, cognitive status with a Mini-Mental State Examination score > 24, at least one month without having been treated with rehabilitation will be included. All subjects must have signed the informed consent.

All subjects who have relapses in the last three months and who have visual deficits that could compromise the use of MS-FIT will be excluded.

**Assessment and Treatment**

For the feasibility study, the assessment will be performed before (PRE) and after (POST) the 6 weeks of treatment. For the RCT, the assessment will be performed before (PRE) and after (POST) the 12 weeks of treatment and 6 weeks after the POST time-point (follow-up, FU).

In both the feasibility and RCT studies two groups (i.e. contro group and experimental group) will be recruited. For the entire duration of the 6 (treatment; feasibility study) or 12 + 6 (treatment + follow-up; RCT) weeks, both groups are required to continue performing normal physical activities that they may already be performing; in addition, the experimental group is required to perform the MS-FIT exercises at least 3 times a week for a total of 30 minutes per session (also distributed during the day with a minimum time slot of 10 minutes) for the 6 weeks in the feasibility study, or the first 12 in the RCT.

The administration of MS-FIT will be self-managed at home by the participant who will trained to the use of the Xbox One and Microsoft Kinect 2.0 platform before the release.

For both groups it will not be possible to perform rehabilitation treatments except for sphincter rehabilitation, speech therapy, and psychological support.

The assignment to the experimental or control group will be carried out according to a random computerized procedure.

Participants in the RCT will have a blood sample taken at the PRE time-point.

**Information collection**

An electronic CRF will be used to collect clinical and demographic data and the results of the tests/questionnaires administered.

The data will be de-identified using a code and will be accessible only to the heads of the research groups of each partner of the project.

**Insurance**

An insurance for the project was stipulated with the company QBE, with policy number 063 0000483, valid from 24:00 on 19/11/2022 to 24:00 on 31/07/2023.

The maximum coverage per subject is 1000000 euros while the aggregate maximum coverage for the entire duration of the Clinical Trial is 7500000 euros.

The Deadline for the manifestation of damages caused to the subjects of the trial is 24 months from 31/07/2023 while the Deadline for the presentation of compensation requests is 36 months from 31/07/2023.

Centers participating in the study.

• Italian Multiple Sclerosis Foundation (FISM), Genoa

• IRCCS Foundation “Carlo Besta” Neurological Institute, Milan

• Institute of Experimental Neurophysiology, San Raffaele Hospital, Milan

• University of Genoa, Genoa

• IRCCS Neuromed, Pozzilli, Isernia

• University of Cagliari, Cagliari

• University of Padua, Padua

• University of Bari, Bari

• IRCCS Don C. Gnocchi Foundation, Milan

• ERICCS Istituto delle Scienze Neurologiche di Bologna, Bologna

• University of Campania, Naples

• S. Camillo-Forlanini Hospital, Rome

• University of Catania, Catania

• IRCCS Centro Neurolesi “Bonino Pulejo”, Messina

**INFORMED CONSENT**

I, the undersigned

SURNAME ____________________________________________ NAME__________________________________________

I AGREE TO PARTICIPATE IN THE STUDY

Patient’s signature _________________________________________ Date ______________________

Doctor’s signature__ _______________________________________________________ Date ______________________
