# Supplementary material for: Effect of an Internet-Based Pilates Telerehabilitation Intervention in People With Multiple Sclerosis: Protocol for a Randomized Controlled Trial
Source: JMIR Res Protoc. 2025 Feb 3;14:e58026. doi: 10.2196/58026 (PMC11833266; doi:10.2196/58026)
Supplement: Multimedia Appendix 3 [file resprot_v14i1e58026_app3.docx]

| ***Type*** | ***Domain*** | ***Measure*** | ***T0*** | ***12 weeks*** | ***T1*** | ***6 weeks*** | ***T2*** |
| --- | --- | --- | --- | --- | --- | --- | --- |
| Performance Measure | Motor function | TUG | x |  | x |  | x |
| Performance Measure | Motor function | T25FW | x |  | x |  | x |
| Performance Measure | Motor function | Ambulation Index | x |  | x |  | x |
| Performance Measure | Motor function | 2MWT | x |  | x |  | x |
| Performance Measure | Motor function | 9HPT | x |  | x |  | x |
| Performance Measure | Cognition | BICAMS (SDMT) | x |  | x |  | x |
| Performance Measure | Cognition | BICAMS (CVLT-II) | x |  | x |  | x |
| Performance Measure | Cognition | BICAMS (BVMT) | x |  | x |  | x |
| Patient Reported Outcome | Motor function | MSWS-12 | x |  | x |  | x |
| Patient Reported Outcome | Motor function | VAS (0-10) Balance | x |  | x |  | x |
| Patient Reported Outcome | Fatigue | MFIS | x |  | x |  | x |
| Patient Reported Outcome | Quality of Life | MSQoL-54 | x |  | x |  | x |
| Patient Reported Outcome | Wellbeing | PWB | x |  | x |  | x |
| Patient Reported Outcome | Physical activity | IPAQ | x |  | x |  | x |
| Patient Reported Outcome | Physical activity | Minnesota LTPAQ | x |  |  |  |  |
| Patient Reported Outcome | Technology | Ad-hoc questionnaire | x |  |  |  | x |
| Patient Reported Outcome | Technology | CSQ-8 |  |  |  |  | x |
| Patient Reported Outcome | Technology | TSQ |  |  |  |  | x |
| Patient Reported Outcome | Intervention effect | PGIC | x |  | x |  | x |
| Blood sample | Genetic | Polymorphisms | x |  |  |  |  |
| Digital biomarker | Adherence | Number of sessions |  | x |  |  |  |
| Event | Adherence | Dropout | x | x | x | x | x |
| Event | Safety | Issues | x | x | x | x | x |
| Clinical information | Treatment | Drugs specific for MS | x | x | x | x | x |
| Clinical information | Treatment | Drugs not specific for MS | x | x | x | x | x |
| Clinical information | Comorbidity | Other medical condition | x | x | x | x | x |
